# Development and evaluation of an evidence-based health information on benzodiazepines and z-drugs

**DOI:** 10.1093/eurpub/ckac131.495

**Published:** 2022-10-25

**Authors:** C Lindemann, J Heeg, J Dirmaier, U Verthein, M Haerter

**Affiliations:** 1 University Medical Center Hamburg-Eppendorf, Institute and Outpatients Clinic of Medical Psychology, Hamburg, Germany; 2 University Medical Center Hamburg-Eppendorf, Centre of Interdisciplinary Addiction Research, University Hamburg, Hamburg, Germany

## Abstract

**Background:**

With approximately 1.1-1.4 million people in Germany taking benzodiazepines (BZDs) and z-drugs in a problematic or dependent manner, this is a relevant public health problem which needs to be adressed. However, it is well known that affected individuals do not receive the counseling or treatment they need. One possible solution is preventive education of affected individuals and medical practioners using evidence-based and target-group-health information (HI).The project EDER-MIA aimed to develop and evaluate evidence-based and target-group-related HI.

**Methods:**

With the help of three different focus groups (18-40-years; women: 40-60-years; > 60-year-olds), the information needs of affected individuals were assessed. Based on the results, we developed the HI and received feedback from health experts. The HI was implemented online (http://www.psychenet.de) and evaluated with the ‘Usefulness Scale of Patient Information’ (USE): An assessment was made on 3 subscales with a 10-point scale from ‘1=disagree at all’ to ‘10=agree completely'. By forming a sum score (range: 0-90), an overall assessment was calculated.

**Results:**

The results of the focus group study revealed the persons taking BZDs or z-drugs were in need about informations about sleep problems, the risk of taking BZDs and z-drugs and a better orientation regarding help services. The evaluation of the health information achieved medium acceptance rates of usefulness for affected persons (N = 192, 68.2% female; mean= 54.3, sd = 15.4). A somewhat higher usefulness was recognised by the medical staff (N = 58, 69.0% female, mean=64.7, sd = 17.2).

**Conclusions:**

The development of target group-specific HI with the participation of affected individuals and experts is suitable for topics that are associated with possible experiences of shame and stigma. In addition to providing information about the risks of taking BZDs and z-drugs, screening of problematic BZD and z-drug use is also a matter of concern.

**Key messages:**

• BZDs and z-drug use is a relevant public health concern which needs to be adressed.

• Target-group specific health information for BZD and z-drug use accompanied by a screening-test can encourage affected individuals to seek medical advice.

